# Personality Traits as Risk Factors for Treatment-Resistant Depression

**DOI:** 10.1371/journal.pone.0063756

**Published:** 2013-05-22

**Authors:** Michio Takahashi, Yukihiko Shirayama, Katsumasa Muneoka, Masatoshi Suzuki, Koichi Sato, Kenji Hashimoto

**Affiliations:** 1 Department of Psychiatry, Teikyo University Chiba Medical Center, Ichihara, Japan; 2 Division of Clinical Neuroscience, Chiba University Center for Forensic Mental Health, Chiba, Japan; Rikagaku Kenkyūsho Brain Science Institute, Japan

## Abstract

**Background:**

The clinical outcome of antidepressant treatment in patients with major depressive disorder (MDD) is thought to be associated with personality traits. A number of studies suggest that depressed patients show high harm avoidance, low self-directedness and cooperativeness, as measured on the Temperament and Character Inventory (TCI). However, the psychology of these patients is not well documented.

**Methods:**

Psychological evaluation using Cloninger’s TCI, was performed on treatment-resistant MDD patients (n = 35), remission MDD patients (n = 31), and age- and gender-matched healthy controls (n = 174).

**Results:**

Treatment-resistant patients demonstrated high scores for harm avoidance, and low scores for reward dependence, self-directedness, and cooperativeness using the TCI, compared with healthy controls and remission patients. Interestingly, patients in remission continued to show significantly high scores for harm avoidance, but not other traits in the TCI compared with controls. Moreover, there was a significant negative correlation between reward dependence and harm avoidance in the treatment-resistant depression cohort, which was absent in the control and remitted depression groups.

**Conclusions:**

This study suggests that low reward dependence and to a lesser extent, low cooperativeness in the TCI may be risk factors for treatment-resistant depression.

## Introduction

Antidepressants are commonly used in the treatment of major depressive disorder (MDD). Between 60 and 70 percent of depressed patients respond to treatment with the first prescribed antidepressant at maximal doses for at least 2 months, and 80 to 90 percent of these patients respond to the first or second choice prescribed antidepressant. The 5 to 15 percent of patients who do not respond to treatment are deemed to have treatment-resistant depression [1]. It is noteworthy that response is defined as a reduction to less than 50 percent in depressive symptoms, but not necessarily recovery. Remission is defined as a full recovery, classified as a score of less than 7 on the Hamilton Rating Scale for Depression (HAM-D) [1]. In order to study the psychopathological aspects of treatment-resistant depression, it is necessary to extract the core features of treatment-resistant depression.

Cloninger and his colleagues developed a dimensional psychosocial model of personality. This Temperament and Character Inventory (TCI) defines four dimensions of temperament: novelty seeking, harm avoidance, reward dependence, and persistence, and three dimensions of character: self-directedness, cooperativeness, and self-transcendence [2]. Personality is considered to affect the outcome of mood disorder. It is well established that depressed patients showed high scores of harm avoidance and that the severity of depression correlates positively with harm avoidance scores on the TCI [3]–[13]. Furthermore, scores in the harm avoidance section are altered by depression and antidepressant treatment [4], [7], [8], [14]. In a meta-analysis of MDD study data, harm avoidance scores showed a clear negative change from baseline to endpoint [15]. Similarly, it is known that depressed patients showed low scores of self-directedness and that the severity of depression correlates negatively with self-directedness scores on the TCI [5]–[13], [16].

Favorable outcomes after antidepressant treatment are associated with personality score changes. Depressive patients with low harm avoidance scores on the TCI tend to have good outcomes [17], [18]. Typical TCI scores indicating a favorable outcome, show decreases in harm avoidance and self-transcendence and increases in self-directedness and cooperativeness, from baseline to post-treatment values [19]. Furthermore, the harm avoidance rate in treatment-resistant patients is significantly higher than that of the treatment-response group [18], [20]. However, no significant personality changes were observed in patients with poor outcome after antidepressant treatment [19]. Both non-responders and responders showed increased harm avoidance scores, and decreased self-directedness and cooperativeness scores on the TCI [7]. However, it remains unclear which components of personality influence treatment-resistance in MDD patients.

The purpose of this study was to evaluate the psychological features of treatment-resistant patients with MDD. Here, we investigated the possibility of personality biases in treatment-resistant patients with MDD, using Cloninger’s seven-factor model, TCI [2].

## Methods

### Ethics Statement

The study was approved by the ethics committee of Teikyo University Chiba Medical Center (study number 09–30) and performed in accordance with the Declaration of Helsinki. Written informed consent was obtained from all participants after the procedures had been fully explained.

### Participants

A total of 174 healthy subjects, 35 antidepressant treatment-resistant depressive patients, and 31 remitted depressive patients were enrolled in this study (**Table 1**). All patients were recruited from the outpatient clinics of Teikyo University Chiba Medical Center (Ichihara, Chiba, Japan), and met the DSM-IV criteria for MDD (first episode) [21]. Two senior-level psychiatrists assessed patients’ psychopathology. Patients were all physically healthy and free of alcohol or drug abuse. Inclusion criteria required symptoms of moderate depression, after treatment with at least two antidepressants, for 8 weeks. Patient scores were 14 or more on the 17-item HAM-D scale, where the definition of remission (recovery) was 7 or less [1]. Healthy control subjects with no past history of psychiatric disorders or drug dependence were recruited. Clinical information regarding the subjects is provided in **Table 1**. The duration of depression in treatment-resistant patients was significantly longer than in remitted depression (**Table 1**).

**Table 1:** Demographic information of subjects.

	Healthy control	Remitted depression	Treatment-resistant depression	P valued
Current age (years)	36.76±9.52 (17–60)	40.58±7.88 (28–55)	38.74±9.42 (22–53)	0.080
Sex (male/female)	135/39	18/13	24/11	0.056
Age on set (years)		38.00±8.42 (25–54)	35.94±8.93 (17–50)	0.366
Duration of depressive state (months)		19.04±24.26 (3–49)	36.46±21.32** (9–98)	<0.001
Duration of treatment (months)		23.31±22.32 (3–103)	30.06±26.23 (4–97)	0.235
HAM-D		4.38±1.63 (2–7)	18.31±4.04** (14–28)	<0.001
Trial numbers of antidepressants		1.20±0.40 (1–2)	2.60±1.56** (2–9)	<0.001

Data are shown as mean ± SD.

Parenthesis is the range.

HAM-D: Hamilton Rating Scale for Depression.

** p<0.001 as compared to the remitted group (Student’s t-test).

### Personality Scores and Psychological Tests

Personality was assessed using the TCI-125 (a shortened version of the TCI) [2], [10], [22], [23]. The Japanese version of TCI has been validated and tested for reliability in Japan [24], [25]. Items were rated on a four-point scale (1; totally disagree, 2; disagree, 3; agree and 4; totally agree). This test covers four dimensions of temperament, namely: harm avoidance, novelty seeking, reward dependence, and persistence, and three dimensions of character: self-directedness, cooperativeness, and self-transcendence. To obtain normative data, this test was performed on the 174 healthy controls.

### Statistical Analysis

Data from the seven TCI dimensions were first analyzed using multiple analysis of variance (MANOVA), to determine the simultaneous existence of significant differences. Statistical differences among the three groups were determined by a one-way factorial analysis of variance (ANOVA), followed by a multiple comparison test (Scheffe’s test). Chi-square test was used for categorical variables. Statistical evaluation between the two groups was performed using a two-tailed Student’s t-test. Coefficients among scores of TCI were estimated by Pearson coefficient. Differences were considered to be significant when p values were less than 0.01.

## Results

**Figure 1:**
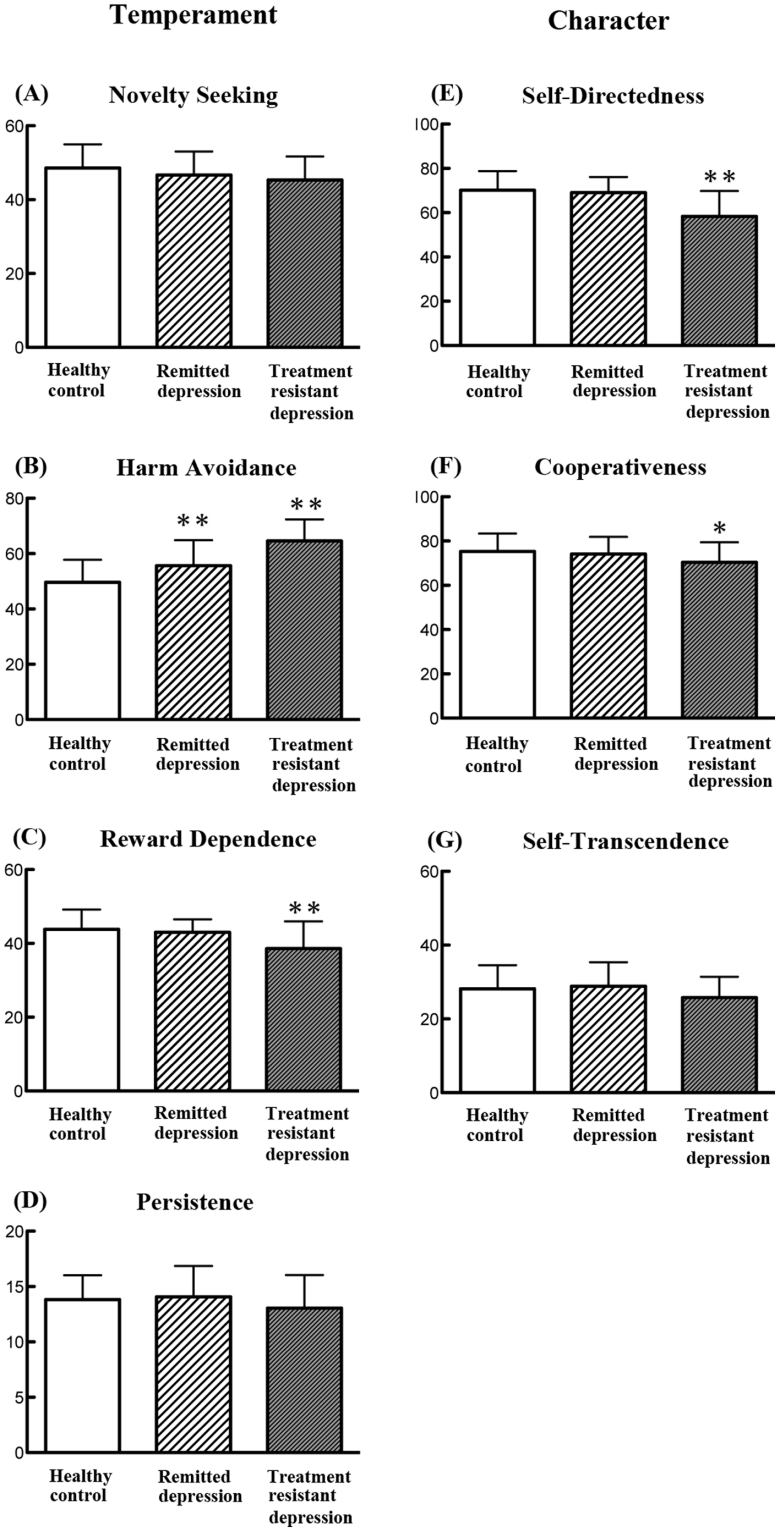
The data of TCI. Analysis of variance (ANOVA) shows a significant difference between three groups for (B) Harm Avoidance (F (2,237) = 50.58; P<0.001), (C) Reword Dependence (F (2,237) = 13.19; P<0.001), (E) Self-Directedness (F (2,237) = 25.98; P<0.001), and (F) Cooperativeness (F 2,237) = 5.42; P = 0.005). There are not significant differences between three groups in (A) Novelty seeking, F (2,237) = 4.44, p = 0.013, (D) Persistence, (F (2,237) = 1.81, P = 0.167, and (G) Self-transcendence, F (2,237) = 2.51, P = 0.084. *P<0.01, **P<0.001 compared to control (ANOVA followed by Scheffe’s test).

MANOVA indicated a significant group effect (F = 9.101, P<0.0001). Subsequent one-way ANOVA demonstrated that treatment-resistant patients showed significantly altered scores on harm avoidance, reward dependence, self-directedness and cooperativeness, but not novelty seeking, persistence, or self-transcendence compared with remitted depression patients and healthy controls (**Figure 1**). Relative to healthy controls, patients in remission only showed significantly increases in scores for harm avoidance (**Figure 1**).

The subscales of each dimension of the TCI are shown in **Table 2**. Treatment-resistant patients showed significantly high scores for anticipatory worry, fear of uncertainty and fatigability in the harm avoidance, and low scores for attachment in reward dependence, responsibility, purposefulness, resourcefulness and congruent second nature in the self-directedness, and empathy, helpfulness and pure-heartedness in the cooperativeness category, compared to remitted depression patients and healthy controls.

**Table 2:** Comparison of TCI subscales in subjects.

	Healthy control	Remitted depression	Treatment-resistant depression	F	P
	(n = 174)	(n = 31)	(n = 35)		
**< Novelty seeking >**	48.58±6.40	46.65±6.35	45.31±6.37	4.44	0.013
Exploratory excitability	12.60±1.96	12.36±2.11	11.49±2.12	4.54	0.012
Impulsiveness	12.43±2.26	11.65±2.32	10.69±2.78**	8.66	<0.001
Extravagance	12.68±2.81	12.45±2.40	12.71±2.86	0.09	0.916
Disorderliness	10.78±2.22	10.19±2.65	10.23±1.82	1.58	0.208
**< Harm avoidance >**	49.62±8.15	55.65±9.16**	64.59±7.81** ^,^ ##	50.58	<0.001
Anticipatory worry	11.79±2.39	13.17±2.40	16.03±2.07** ^,^ ##	48.70	<0.001
Fear of uncertainty	13.98±2.34	14.78±2.94	16.67±2.17** ^,^ #	18.65	<0.001
Shyness	11.97±2.96	13.45±2.95	14.97±3.11**	16.25	<0.001
Fatigability	11.92±2.60	14.26±2.61**	16.94±2.24** ^,^ ##	61.32	<0.001
**< Reward dependence >**	43.82±5.35	43.00±3.51	38.60±7.36** ^,^ #	13.19	<0.001
Sentimentality	14.23±2.17	14.32±2.24	13.00±2.99	4.36	0.014
Attachment	14.39±2.67	14.07±2.00	11.29±3.55** ^,^ ##	18.64	<0.001
Dependence	15.17±2.26	14.61±1.63	14.34±2.95	2.31	0.102
**<Persistence >**	13.83±2.20	14.07±2.79	13.06±2.98	1.81	0.167
**< Self-directedness >**	70.16±8.64	69.03±7.06	58.28±11.51** ^,^ ##	25.98	<0.001
Responsibility	15.79±2.41	13.25±2.98	13.26±2.98**	16.60	<0.001
Purposefulness	14.34±1.93	14.03±2.09	11.14±2.84** ^,^ ##	33.81	<0.001
Resourcefulness	14.21±2.07	13.42±2.55	11.14±2.57** ^,^ ##	28.14	<0.001
Self-acceptance	11.91±3.46	12.90±3.24	11.17±3.86	2.03	0.134
Congruent second nature	13.83±2.00	14.00±1.97	11.51±2.23** ^,^ ##	19.93	<0.001
**< Cooperativeness >**	75.33±8.03	74.10±7.81	70.37±9.07*	5.42	0.005
Social acceptance	15.83±2.33	15.00±2.56	14.66±2.29	4.56	0.011
Empathy	13.22±1.87	12.90±2.21	11.97±2.27*	5.84	0.003
Helpfulness	15.23±2.07	14.74±2.00	13.37±2.77**	10.66	<0.001
Compassion	15.11±2.73	16.07±2.63	15.66±2.63	1.96	0.143
Pure-heartedness	15.95±1.93	15.39±2.00	14.77±2.39*	5.42	0.005
**< Self-transcendence >**	28.18±6.38	29.61±7.49	25.80±5.65	3.09	0.047
Self-forgetfulness	8.62±2.59	9.23±2.68	8.20±2.69	1.28	0.279
Transpersonal identification	10.03±2.41	10.29±2.82	8.80±2.11	4.23	0.017
Spiritual acceptance	9.65±2.42	10.07±2.63	8.89±2.35	2.59	0.125

Data are shown as mean ± SD.

* p<0.01,

** p<0.001 compared to control (ANOVA followed by Scheffe’s test).

# p<0.01,

## p<0.001 compared to remitted depression (ANOVA followed by Scheffe’s test).

Interestingly, harm avoidance, self-directedness and self-transcendence correlate significantly with HAM-D scores in all MDD patients (harm avoidance, r = 0.434, p<0.0001; self-directedness, r = −0.485, p<0.0001; self-transcendence, r = −0.343, p<0.001). In contrast, there were no correlations between TCI scores and severity of depression in patients with treatment-resistant depression (data not shown).

There was a significant negative correlation between reward dependence and harm avoidance in treatment-resistant depression patients. This correlation was not present in healthy controls and remitted depression patients (**Table 3**). Furthermore, there was a significant negative relationship between cooperativeness and the dimensions of novelty seeking and harm avoidance, in the treatment-resistant group, but not in the healthy control and remitted depression groups (**Table 3**). Conversely, there was a significant negative correlation between novelty seeking and harm avoidance scores in the healthy control and remitted depression groups, but not in the treatment-resistant depression group (**Table 3**).

**Table 3:** Correlates of TCI variables.

Healthy control (n = 174)	NS	HA	RD	P	SD	C	ST
Novelty seeking (NS)	–						
Harm avoidance (HA)	.463**	–					
Reward dependence (RD)	.063	−.115	–				
Persistence (P)	−.018	−.146	.109	–			
Self-directedness (SD)	−.142	−.399**	232*	.093	–		
Cooperativeness (C)	−.093	−.169	.639**	.177	.358**	–	
Self-transcendence (ST)	.194	−.043	.007	.148	−.308**	.041	–
**Remitted depression (n = 31)**	**NS**	**HA**	**RD**	**P**	**SD**	**C**	**ST**
Novelty seeking (NS)	–						
Harm avoidance (HA)	−.525*	–					
Reward dependence (RD)	.376	−.173	–				
Persistence (P)	.287	−.401	.252	–			
Self-directedness (SD)	.292	−.334	.466*	.027	–		
Cooperativeness (C)	.071	−.055	.486*	−.069	.396	–	
Self-transcendence (ST)	.438	−.288	.383	.263	.105	.314	–
**Treatment – resistant depression (n = 35)**	**NS**	**HA**	**RD**	**P**	**SD**	**C**	**ST**
Novelty seeking (NS)	–						
Harm avoidance (HA)	.034	–					
Reward dependence (RD)	−.074	−.466*	–				
Persistence (P)	−.246	−.366	.330	–			
Self-directedness (SD)	−.407	−.603**	.512*	.290	–		
Cooperativeness (C)	−.437*	−.519*	.599**	.368	.577**	–	
Self-transcendence (ST)	.377	−.121	.119	.295	−.023	.121	–

* p<0.01,

** p<0.001.

## Discussion

In this study, we found a number of psychological features that appeared to be associated with treatment-resistant MDD. Firstly, we found treatment-resistant patients showed higher scores for harm avoidance and lower scores for self-directedness on the TCI, consistent with previous reports on depressed patients [5], [6], [9]–[11], [13]. We also found that harm avoidance and self-directedness correlate significantly with HAM-D scores in both remitted and treatment-resistant patients with depression, replicating previous studies [6], [7], [9], [11], [12]. Of the seven published studies on TCI scores in depression (**Table 4**), all found significant alterations in the score for harm avoidance and all but one in the score for self-directedness, indicating that this is a common pattern in depression. Since treatment-resistant patients suffer from depressive symptoms, it is not surprising that these non-responders showed the same pattern of high harm avoidance and low self-directedness as depressed patients.

**Table 4:** Summary of TCI scores of depressed patients.

	NS	HA	RD	P	SD	C	ST
Hanssenne et al, 1999 [5]	−	↑	−	−	↓	↓	↑
Richiter et al, 2000 [6]	↓	↑	−	↓	↓	−	−
Farmer et al, 2003 [9]	↓	↑	−	−	↓	↓	−
Smith et al, 2005 [10]	−	↑	−	−	↓	−	−
Celikel et al, 2009 [11]	−	↑	−	−	↓	−	−
Sasayama et al, 2011 [13]	↑	↑	−	−	↓	↓	−
Kampman et al, 2012 [18]	−	↑	↑	−	−	−	−
This study (treatment-resistant)	−	↑	↓	−	↓	↓	−

NS: Novelty seeking, HA: Harm avoidance, RD: Reward dependence, P: Persistence, SD: Self-directedness, C: Cooperativeness, ST: Self-transcendence.

↑: Increase, ↓: Decrease, −: No change.

Secondly, treatment-resistant patients demonstrated low scores for reward dependence on the TCI. To the best of our knowledge, this is the first report showing this feature in treatment-resistant MDD patients, and there are no equivalent reports in depressed patients groups (**Table 4**). This is suggestive of low reward dependence being a characteristic feature of treatment-resistant MDD. Looking more closely at reward dependence, the subscale altered between remitted patients and healthy controls is attachment. Thus, it is likely that the dimension of attachment in reward dependence could be specific to treatment-resistant patients. In a recent study of the antidepressant treatment responders, scores for reward dependence had a small overall positive change from baseline to endpoint [15]. This is supportive of other studies where depressive patients showing high reward dependence on the TCI, also showed a good outcome after antidepressant treatment [17], [22]. Interestingly, scores for reward dependence in non-depressive siblings of depressed patients, were significantly higher than for siblings with a history of depression, suggesting that high reward dependence may protect against the development of depression [9]. However, it is unknown whether the enduring characteristics of non-responders are primary or secondary to the disease. A long history of treatment-resistant depression may induce character changes within patients and these changes may persist after the recovery from disease. Future studies will be needed to elucidate these points.

Thirdly, treatment-resistant patients showed low scores for cooperativeness. Significant scores for low cooperativeness in depressed patients were reported in three of the seven studies examining this issue (**Table 4**) [5], [9], [13]. This lower concordance suggests that low cooperativeness is a less common characteristic of depressed patients compared with high harm avoidance and low self-directedness. Within the subscale of cooperativeness, the most significantly altered dimensions in comparison with healthy controls, were empathy, helpfulness and pure-heartedness. These results should prove useful in tailoring psychotherapy for MDD treatment-resistant patients. The cooperativeness score correlated negatively with the severity of depression among depressive patients [5]. Other studies showed large increases in cooperativeness and self-directedness scores among treatment responders in MDD, with relative stability of these features among non-responders during treatment [7], [19], [23]. A recent study demonstrated that cooperativeness was strongly associated with perceived social support [26]. As mentioned before, it remains unknown whether low cooperativeness is related primarily to treatment-resistant depression or is a secondary effect due to the long duration of illness. Previous studies using TCI showed that low scores for cooperativeness and self-directedness strongly predicted personality disorders in patients with mood disorders [16], [23]. Furthermore, low cooperativeness could be a predictor for hostility and paranoia [27]. Low reward dependence is strongly associated with cluster A symptoms, such as paranoid, schizoid and schizotypal personality disorders [16]. It is well known that personality disorders have negative effects on the course and outcome of MDD [28]–[30]. Therefore, treatment-resistant patients with MDD may suffer from some underlying personality disorder traits, although patients with overt personality disorders were excluded from this study at recruitment.

Fourthly, our results showed significant negative correlations between reward dependence and harm avoidance and between cooperativeness and novelty seeking in the treatment-resistant depression group, which were absent in healthy controls and remitted depression patients. This newly highlighted relationship in treatment-resistant depression patients indicates that low scores for reward dependence and cooperativeness could at least in part be due to harm avoidance and novelty seeking, respectively.

Finally, patients in remission still showed high scores for harm avoidance, compared with normal controls, although the difference was small. In this case, the altered subscale between remitted patients and controls was fatigability. This finding is supported by a previous study demonstrating that even though a significant reduction occurred, higher harm avoidance among unipolar depression patients persisted after treatment, compared with healthy controls [6]. Future studies will be needed to elucidate whether harm avoidance plays a role in the relapse of depression.

In conclusion, treatment-resistant patients with MDD demonstrated high scores for harm avoidance, and low scores for reward dependence, self-directedness, and cooperativeness, using the TCI. It is well known that depressed patients show high harm avoidance and low self-directedness, and sometimes low cooperativeness on the TCI. Patients with treatment-resistant MDD show persistent symptoms of depression. Our findings suggest that low reward dependence and to a lesser extent, cooperativeness on the TCI may constitute possible risk factors of treatment-resistant depression.
